# Differences of Clinicopathological Features between Metaplastic Breast Carcinoma and Nonspecific Invasive Breast Carcinoma and Prognostic Profile of Metaplastic Breast Carcinoma

**DOI:** 10.1155/2022/2500594

**Published:** 2022-08-22

**Authors:** Yue Qiu, Yuhui Chen, Li Zhu, Hongye Chen, Yongjing Dai, Baoshi Bao, Lin Tian, Xiaopeng Hao, Jiandong Wang

**Affiliations:** ^1^Chinese PLA Medical School, Beijing 100853, China; ^2^Department of General Surgery, The First Medical Center, Chinese PLA General Hospital, Beijing 100853, China

## Abstract

**Introduction:**

Metaplastic breast carcinoma is a rare special type of breast cancer, which has distinguished clinical characteristics. We aimed to evaluate the clinicopathological features of metaplastic breast carcinoma compared with nonspecific invasive breast carcinoma and study the prognosis of metaplastic breast carcinoma.

**Methods:**

We reviewed metaplastic breast carcinoma cases (*n* = 37) from January 2000 to December 2021 and nonspecific invasive breast carcinoma cases (*n* = 433) from January 2019 to December 2020 extracted from our institution retrospectively. The following variables were recorded, including the patients' general information, complications, T stage, expression of estrogen receptor, progesterone receptor, human epidermal growth factor receptor 2, Ki-67, molecular subtyping, lymph node status, skin or chest wall involvement, vessel carcinoma embolus, therapy modality (surgical treatments, chemotherapy, and radiotherapy), and survival.

**Results:**

Patients with metaplastic breast carcinoma had more advanced disease than patients with nonspecific invasive breast carcinoma (T stage: *P*=0.0011). A greater proportion of metaplastic breast carcinoma presented with triple-negative breast cancer than nonspecific invasive breast carcinoma (79.41% vs. 12.47%, *P* ≤ 0.001). Our study showed that the skin or chest wall invasion was more frequent in metaplastic breast carcinoma patients (11.76% vs. 1.62%, *P*=0.005). The 5-year survival rate for metaplastic breast carcinoma patients was 57.66% (95% CI: 0.3195∼0.7667). No local recurrence was observed while distant metastasis occurred in 33.33% of patients with metaplastic breast carcinoma. Death due to disease occurred in 24.24% of patients with metaplastic breast carcinoma.

**Conclusion:**

The majority of metaplastic breast carcinoma patients had more advanced disease and triple-negative disease than nonspecific invasive breast carcinoma patients. Also, metaplastic breast carcinoma patients had frequent skin or chest wall invasion and a high rate of distant metastasis and mortality.

## 1. Introduction

Metaplastic breast cancer (MBC) is a rare group of breast cancers, accounting for about 0.25%∼1.00% of all breast cancers [1]. MBC is composed of a mixed group of tumors showing divergent differentiation patterns. Up to now, the prognosis and treatment of MBC are overall indefinite, and evidence suggests that it has distinguished characteristics compared with nonspecific invasive breast cancer (NSIBC) [2, 3]. In previous studies, this disease has a high T stage, negative hormone receptors, and low Her-2 expression, and the incidence of local recurrence and distant metastasis varies with its pathologic classifications [4]. Consequently, clinicians and pathologists attempt to have increased cognizance of MBC.

Due to its rarity, the study connecting with its clinicopathological features is still lacking, prompting this investigation. In this study, we will discuss the disparate clinicopathological characteristics of MBC contrasted with NSIBC and analyze the treatment options and prognosis of this rare type of breast cancer.

## 2. Methods

### 2.1. Data Collection

The data of 37 patients with MBC from January 2000 to December 2021 and 433 patients with NSIBC from January 2019 to December 2020 were collected. Male patients were not included in our study. All procedures performed in this study involving human participants were in accordance with the Declaration of Helsinki (as revised in 2013). The study was approved by the Ethics Board of the Chinese PLA General Hospital (NO. S2022-051). Individual consent for this retrospective analysis was waived.

### 2.2. Inclusion and Exclusion Criteria

Inclusion criteria were (1) patients aged between 18 and 75 years old; (2) females; (3) patients with clear pathological diagnosis after surgery performed in our hospital; (4) a comprehensive medical history. Exclusion criteria were (1) bilateral breast cancer; (2) recurrent breast cancer; (3) metastatic breast cancer; (4) radiotherapy, chemotherapy, or endocrine therapy have been performed in another hospital before surgery; (5) metaplastic carcinoma from the areola, nipple, and appendages of the skin.

### 2.3. Study Variables

The data of 35 patients with MBC and 433 patients with NSIBC were retrospectively analyzed. The clinicopathological features included age, body mass index (BMI), lesion location, smoking history, drinking history, family history, menopausal status, complications, T stage, the level of estrogen receptor (ER), the progesterone receptor (PR), human epidermal growth factor receptor 2 (Her-2) and Ki-67, molecular classification, lymph node status, the skin or chest invasion rate, vessel carcinoma embolus, and therapy modality (surgical treatments, chemotherapy, and radiotherapy). At the same time, the prognosis of MBC patients was analyzed by follow-up data and the end point of the follow-up was disease-specific survival (DSS). According to the guidelines of the Chinese Society of Clinical Oncology in 2020 [5], the minimum positive threshold of ER, PR, and Ki-67 were 1%, 1%, and 14%, respectively, and Her-2 (3+) or ISH positivity meant Her-2 positivity. Breast cancer was divided into luminal A (ER/PR positive, Her-2 negative with low Ki-67 index) disease, luminal B (ER/PR positive, Her-2 negative with a high Ki-67 index, or ER/PR positive, Her-2 positive) disease, Her-2 positive (ER and PR negative, Her-2 positive) disease, and triple-negative (ER, PR, and Her-2 negative) disease according to molecular subtyping.

### 2.4. Statistical Analysis

All statistical analyses were performed using Stata Statistical Software Version 15.1 (College Station, TX: StataCorp LLC). The Mann–Whitney *U* test was applied in the comparison of the measurement data and ranked data between the two groups. Enumeration data were analyzed by the Pearson *X*^2^ test. Kaplan–Meier analysis was performed to evaluate DSS for MBC. Two-tailed *P* < 0.05 was considered statistically significant.

## 3. Results

### 3.1. General Characteristics of MBC and NSIBC

There was a barely detectable statistically significant difference in the site of the lesion between the two groups (64.71% vs. 48.27%, *P*=0.065). But no distinction was found in other general information between the two groups (*P* > 0.05). Also, there was no difference in complications between the two groups (*P*=0.310, *P*=1.000, and *P*=1.000, respectively). However, patients with MBC were significantly higher in the T stage than NSIBC (*P*=0.0011). The rate of skin or chest wall invasion in patients with MBC was significantly higher than that in patients with NSIBC (11.76% vs. 1.62%, *P*=0.005). As for lymph node status, vessel carcinoma embolus, and surgical treatments, features displayed in MBC were not distinguished (*P*=0.826, *P*=0.447, and *P*=0.970, respectively). (see Table 1).

### 3.2. Comparison of Pathological Patterns between the Two Groups

The rate of ER positivity and PR positivity of MBC was lower than that of NSIBC (*P* ≤ 0.001). But there was no significant difference in the level of Ki-67 and Her-2 between the two groups (*P*=1.000, and *P*=0.135, respectively). The majority of MBC patients were triple-negative compared to NSIBC (79.41% vs. 12.47%, *P* ≤ 0.001) (Table 2).

### 3.3. Histopathological Types of MBC

**Figure 1:**
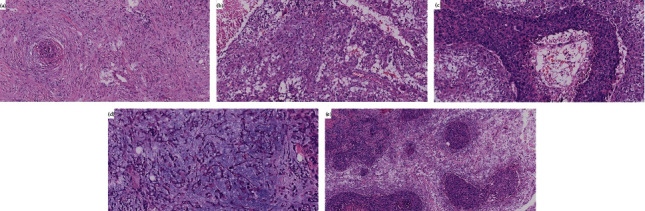
Photomicrographs of MBC. (a). Fibromatosis-like metaplastic carcinoma 200×. (b) Spindle cell carcinoma 200×. (c) Squamous cell carcinoma 200×. (d) Metaplastic carcinoma with mesenchymal differentiation 200×. (e) Mixed metaplastic carcinoma 100×. MBC: metaplastic breast carcinoma.

There were seventeen cases (50.00%) of squamous cell carcinoma, one case (2.94%) of spindle cell carcinoma, eleven cases (32.35%) of carcinoma with mesenchymal differentiation, four cases (11.76%) of mixed metaplastic carcinoma, and one case (2.94%) of fibromatosis-like metaplastic carcinoma (Table 3, Figure 1).

### 3.4. Survival of MBC

**Figure 2:**
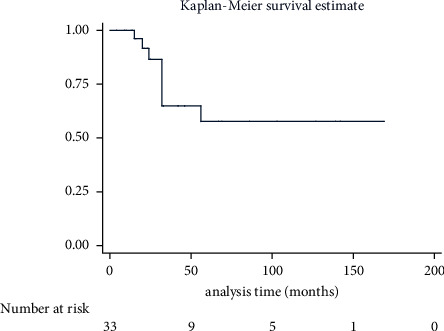
Disease-specific survival for 33 cases of MBC patients. A total of 37 MBC patients were followed up, and 33 of them were able to collect complete information. The number at risk at the time point was presented. MBC: metaplastic breast carcinoma.

**Figure 3:**
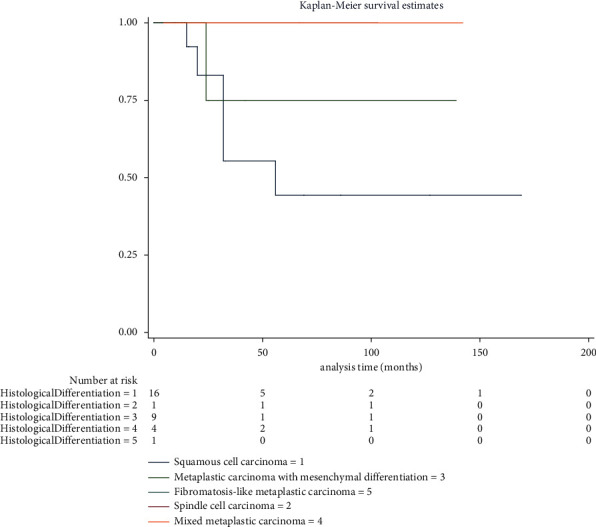
Disease-specific survival for 31 cases of MBC patients with different histological differentiation. A total of 37 MBC patients were followed up, and 31 of them were able to collect complete information. The number at risk at the time point was presented. MBC: metaplastic breast carcinoma.

The median follow-up time was 25.00 (15.00∼56.00) months. The 5-year survival rate of MBC patients was 57.66% (95% CI: 0.3195∼0.7667). There were no cases (0.00%) of recurrence, ten cases (33.33%) of distant metastasis, and eight cases (24.24%) of death due to MBC (Table 4). The DSS curve for MBC is displayed in Figure 2. No survival difference was observed between MBC patients with different pathologic patterns (*X*^2^ = 2.71, *P*=0.6068) (Figure 3).

## 4. Discussion

MBC, which is distinguished from other types of breast cancer, is a rare type of breast cancer accounting for less than 1.00% of invasive breast cancer [1, 6]. The results of our study showed that patients with MBC had a high T stage, low ER, PR, and Her-2 positivity, as well as quite a few triple-negative diseases, a high skin or chest wall invasion rate, a high distant metastasis rate, and high mortality.

In consideration of the World Health Organization classification of breast tumors, seven types of MBC are presented here: low-grade adenosquamous carcinoma, fibromatosis-like metaplastic carcinoma, spindle cell carcinoma, squamous cell carcinoma, metaplastic carcinoma with mesenchymal differentiation (chondroid, osseous, and other types of mesenchymal differentiation), mixed metaplastic carcinoma, and myoepithelial carcinoma [7, 8]. MBC is defined as pure and mixed metaplastic breast cancer which occurred usually with invasive ductal carcinoma, and due to this heterogeneity, MBC patients are often misdiagnosed [9]. In our study, a total of 34 patients with MBC were able to obtain complete pathological diagnoses, including 17 cases (50.00%) of squamous cell carcinoma, 1 case (2.94%) of spindle cell carcinoma, 11 cases (32.35%) of carcinoma with mesenchymal differentiation, 4 cases (11.76%) of mixed metaplastic carcinoma, and 1 case (2.94%) of fibromatosis-like metaplastic carcinoma. As for biological behavior, MBC is poorly differentiated and greatly aggressive [10]. Previous studies showed that MBC mainly metastasized via the lymphatics and blood, and early hematogenous metastasis often involved the lung and the bone, especially in metaplastic carcinoma with mesenchymal differentiation [11]. There were 11 cases of carcinoma with mesenchymal differentiation, of which 9 cases could obtain follow-up data. One case of them had multiple bone metastases at the time of diagnosis, and one case who had received operation suffered from a distant disease and died of MBC. It meant that the metastasis rate and mortality of MBC were high, which was in accord with earlier research. MBC patients are usually hormone receptor-negative and do not show Her-2 overexpression; that is, the molecular subtyping is triple-negative [1]. The results of our study also verified that MBC patients were mainly triple-negative (79.41%). Compared with triple-negative breast cancer (TNBC) or other types of breast cancer, MBC is characterized by a higher T stage, less lymph node involvement and lymphatic vascular invasion, and higher pathological grade [12], but the possibility of lymph node involvement varies with histopathological subtypes [13, 14]. Our study illustrated that patients in the MBC group expressed a higher T stage and more skin or chest wall invasion compared with patients in the NSIBC group as well. But there was no significant difference in lymph node metastasis and vessel carcinoma embolus between the two groups (*P*=0.826 and *P*=0.447, respectively).

As a separate group, the prognosis of the special type of TNBC was worse than that of the nonspecific type of TNBC, and the histological subtypes had a special prognostic value [15]. These studies showed that MBC was more likely to recur than TNBC and that more patients died of MBC than TNBC. In multivariate analysis, the local recurrence risk of MBC is about twice that of TNBC, and the DSS and overall survival of MBC are worse than TNBC [16–19]. We followed 37 MBC patients, 33 of them were able to collect complete information, and 1 of them was in stage IV at the time of diagnosis with multiple bone metastases. The 5-year survival rate for MBC patients was 57.66% (95% CI: 0.3195∼0.7667). There were 0 cases (0.00%) of recurrence, 10 cases (33.33%) of distant metastasis, and 8 cases (24.24%) of death due to MBC. To further investigate the survival difference between MBC patients with different histological differentiation, we grouped them by histopathological types to analyze survival data. Finally, no significant difference in survival was found in MBC patients with various histopathological types. Certain studies demonstrated that mixed and matrix producing MBC had high recurrence rates [20]. But no remarkable correlation was noted between the histological subtype and local or distant disease control by univariate analysis [4]. Perhaps the low prevalence of MBC limits its further research.

Compared with TNBC, MBC owns a more aggressive disease and may require more intensive therapy [21]. MBC has poor response to neoadjuvant chemotherapy, and the pathological complete response rate is lower than TNBC [22]. There were 5 patients with MBC receiving neoadjuvant chemotherapy, but no patient achieved pathological complete response ultimately. Most MBC patients tend to receive mastectomy and sentinel lymph node biopsy, choosing radiotherapy and chemotherapy as adjuvant treatments. No difference was observed between patients receiving mastectomy and those who underwent lumpectomy [23]. In our study, all patients received operations, including 27 cases of modified radical mastectomy, 3 cases of mastectomy only, and 7 cases of breast-conserving surgery. Because of the advanced disease of MBC patients, the probability of breast-conserving surgery is lower than that of NSIBC, and a high proportion of MBC patients received adjuvant chemotherapy [16, 24]. Meanwhile, MBC has a poor response to conventional chemotherapy, which may be related to the histological diversity and tumor heterogeneity caused by complex tumor genetics [9, 25]. But multivariate analysis showed that postoperative adjuvant radiotherapy was related to better prognosis [26].

There are some possible limitations in our study. This study was a retrospective clinical study, so the information acquired from the materials left by patients seemed inadequate. Also, we failed to explore the feature of risk factors in connection with this disease. The number of cases was not sufficient, and we need a large-scale study to validate these findings. In addition, some patients did not have enough follow-up time, and the survival analysis was deficient.

## 5. Conclusion

MBC is a rare and special type of breast cancer. Compared with NSIBC, MBC is characterized by a higher T stage, lower positivity of ER, PR, and Her-2, more triple-negative disease, a higher rate of skin or chest wall invasion, a higher distant metastasis rate, and mortality. At present, there is extremely lacking studies on MBC, and some compelling evidence is needed to guide clinical practice. It is urgent to conduct research studies on a large scale so as to correctly diagnose the disease, formulate a standardized and effective therapeutic regimen, and achieve a better prognosis.

## Figures and Tables

**Table 1:** General characteristics of MBC and NSIBC.

Category	NSIBC (*n* = 433)	MBC (*n* = 34^a^)	*X * ^2^	*P*-value
Median age in years (IQR)	50.00 (44.00∼56.00)	49.50 (44.00∼56.00)	0.005	0.9963
BMI (kg/m^2^, IQR)	24.00 (22.20∼26.30)	24.15 (22.40∼26.70)	0.354	0.7235

Site of lesion				
Left	209.00 (48.27%)	22.00 (64.71%)	3.4077	0.065
Right	224.00 (51.73%)	12.00 (35.29%)		
Smoking history	5.00 (1.15%)	0.00 (0.00%)	—	1.000
Drinking history	6.00 (1.39%)	1.00 (2.94%)	—	0.413
Family history	21.00 (4.85%)	1.00 (2.94%)	—	1.000
Menopause	184.00 (42.49%)	12.00 (35.29%)	0.6710	0.413
Hypertension	66.00 (15.24%)	3.00 (8.82%)	1.0315	0.310
Diabetes	20.00 (4.62%)	1.00 (2.94%)	—	1.000
Hyperlipidemia	62.00 (14.32%)	4.00 (11.76%)	—	1.000

T Category of the primary tumor (AJCC 8th^b^)				
T1	235.00 (54.27%)	10.00 (29.41%)	3.254	0.0011
T2	176.00 (40.65%)	18.00 (52.94%)		
T3	15.00 (3.46%)	2.00 (5.88%)		
T4	7.00 (1.62%)	4.00 (11.76%)		
Lymph node status	199.00 (46.06%)	15.00 (44.12%)	0.0481	0.826
Skin or chest wall invasion	7.00 (1.62%)	4.00 (11.76%)	—	0.005
Vessel carcinoma embolus	87.00 (20.09%)	5.00 (14.71%)	0.5782	0.447

Surgical treatment^c^				
Lumpectomy	83.00 (19.17%)	7.00 (18.92%)	0.0014	0.970
Mastectomy	350.00 (80.83%)	30.00 (81.08%)		

a. Among 37 cases of MBC, 34 cases had complete medical history materials. b. The 8^th^ version of the America Joint Committee on Cancer (AJCC) staging system. c. In 37 cases of MBC, all cases received surgical treatments. MBC: metaplastic breast carcinoma; NSIBC: nonspecific invasive breast carcinoma; BMI: body mass index; IQR: interquartile range.

**Table 2:** Clinicopathological features of MBC and NSIBC.

Category	NSIBC (*n* = 433)	MBC (*n* = 34^a^)	*X* ^2^	*P* value
Immunohistochemistry				
ER positive	324.00 (74.83%)	3.00 (8.82%)	65.4242	≤0.001
PR positive	301.00 (69.52%)	2.00 (5.88%)	56.0218	≤0.001
Ki-67 positive	372.00 (85.91%)	28.00 (82.35%)	—	1.000
Her-2 positive	117.00 (27.02%)	5^b^ (15.15%)	2.2352	0.135

Molecular subtyping^c^				
Luminal A	54.00 (12.47%)	0.00 (0.00%)	8.242	≤0.001
Luminal B	282.00 (65.13%)	4.00 (11.76%)		
Her-2 positive	43.00 (9.93%)	3.00 (8.82%)		
Triple-negative	54.00 (12.47%)	27.00 (79.41%)		

a. Among 37 cases of MBC, 34 cases had complete medical history materials. b. In 37 cases of MBC, 33 cases could obtain the level of Her-2, and 34 cases could obtain the expression levels of ER, PR, and Ki-67. c. According to the guidelines of the Chinese Society of Clinical Oncology in 2020, the minimum positive threshold of ER, PR, and Ki-67 were 1%, 1%, and 14%, respectively, and Her-2 (3+) or ISH positivity meant Her-2 positivity. Breast cancer was divided into luminal A (ER/PR positive, Her-2 negative with low Ki-67 index) disease, luminal B (ER/PR positive, Her-2 negative with high Ki-67 index, or ER/PR positive and Her-2 positive) disease, Her-2 positive (ER and PR negative and Her-2 positive) disease, and triple-negative (ER, PR, and Her-2 negative) disease according to molecular subtyping. MBC: metaplastic breast carcinoma; NSIBC: nonspecific invasive breast carcinoma; ER: estrogen receptor; PR: progesterone receptor; Her-2: human epidermal growth factor receptor 2.

**Table 3:** Histopathological types of MBC.

Category	MBC (*n* = 34^a^)
Squamous cell carcinoma	17.00 (50.00%)
Spindle cell carcinoma	1.00 (2.94%)
Metaplastic carcinoma with mesenchymal differentiation	11.00 (32.35%)
Mixed metaplastic carcinoma	4.00 (11.76%)
Fibromatosis-like metaplastic carcinoma	1.00 (2.94%)

a. In this study, 34 patients with MBC were able to obtain the pathological materials. MBC: metaplastic breast carcinoma.

**Table 4:** Follow-up of MBC.

Category	MBC (*n* = 33^a^)
Local recurrence	0.00 (0.00%)
Distant metastasis	11.00 (33.33%)
Death	8.00^b^ (24.24%)
Median follow-up time (month, IQR)	25.00 (15.00∼56.00)
5-year survival rate	57.66%

a. A total of 37 MBC patients were followed up, 33 of them were able to collect complete information, and 1 of them was in stage IV at the time of diagnosis. b. All 8 patients died of MBC. MBC: metaplastic breast carcinoma; IQR: interquartile range.

## Data Availability

The data used to support the findings of this study are available from the corresponding author upon request.
